# The Bacteriology of Acute Poliomyelitis

**Published:** 1909-03-13

**Authors:** 


### NEUROLOGY.

### THE BACTERIOLOGY OF ACUTE POLIOMYELITIS.

THE disease known as acute anterior poliomyelitis has received considerable attention of late, and much new light has been thrown upon its nature, notably by Geirsvold* in Norway, and by Dr. Pasteur,† Dr. Foulerton and Dr. MacCormac‡ in this country. It seems likely that a new name will have to be devised for it some day, for the acute inflammatory changes in the anterior cornua of the spinal cord, although a common complication of the disease, are not absolutely essential. At any rate, it is possible for two patients to suffer from the febrile disease simultaneously, and yet for one to exhibit paralysis and the other none: not the residual deformity to which the name Infantile Paralysis is usually given, but the acute paresis or paralysis that is so well described in the text-book accounts of acute anterior poliomyelitis.

It may sound strange that acute anterior poliomyelitis may occur without any paralysis; for hitherto it has only been by the development of the characteristic paralytic symptoms, transient in some cases and more permanent in others, that the disease has been recognised at all. The fact is that a name is required for a specific infective disease, of which acute poliomyelitis is a frequent and prominent but not essential feature. The name acute anterior poliomyelitis has been applied to two entirely different things—a pathological process in the anterior cornua of the spinal cord upon the one hand, and a febrile illness which commonly gives rise to this pathological process upon the other.

For the sake of argument we will suppose that the febrile illness is temporarily termed *Geirsvold's fever*; this may occur without the patient exhibiting any paralysis at all; but when paralysis does arise as a result of that fever, it is due to inflammatory changes in and around the anterior cornual cells of the spinal cord, *i.e.* to acute anterior poliomyelitis.

This opens up an entirely new aspect of the disease; perhaps this will be made even clearer if post-diphtheritic neuritis is used as a comparison. The common form of diphtherial paralysis as it affects the soft palate is a very remarkable clinical picture, but it is not in itself a disease. If, however, diphteria were an obscure febricula, difficult of recognition, it is conceivable that "acute temporary paralysis of the palate" might have been regarded as a special disease just as "acute anterior poliomyelitis" has been hitherto. Acute temporary paralysis of the palate is only one of the complications or sequelæ of a more general febrile disease; the disease—diphtheria—is far commoner than this particular sequela is; very possibly acute temporary paralysis of the palate may have other causes besides diphtheria. Correspondingly acute anterior poliomyelitis is only one of the complications or sequelæ of a more general febrile disease; the disease—here, for convenience, termed "Geirsvold's fever"—is probably commoner than this particular sequela is; and very possibly acute anterior poliomyelitis may have other causes besides Geirsvold's fever.

The grounds upon which this new view of the subject have been based are many, but the two chief are epidemiological and bacteriological. It has been known for some time that this disease sometimes occurs in small epidemics. Dr. Pasteur has recorded a typical example of the family outbreak in which an entire family of seven children was attacked by a mild febrile disorder which was followed in five to seven days by marked motor paralysis in three cases, by muscular tremors and temporary strabismus in another case, and by general muscular tremors in a fifth. It is clear that the nature of the illness in this fifth case and in the two in whom there were no muscle symptoms at all would not have been correctly diagnosed had there not been the other four to guide one. Similar family outbreaks have been recorded by others, and the account of some of these given by Dr. Pasteur is extremely interesting. He gives Geirsvold the credit of having studied the disease most successfully from the epidemiological and bacteriological points of view. This observer traced the disease as it spread from farm to farm in Norway, and he observed that both before and during a local outbreak of typical cases with paralysis there were other cases which were apparently milder or abortive, or at any rate exhibited no paralysis. In these cases there was obvious illness lasting from one to four days, the principal symptoms being fever with rigors, sore throat, sweating, and sometimes evidence of irritation of the central nervous system. As connecting links between typical paralytic cases and those in which there was no paralysis at all, there were intermediate cases in which a progressive paralysis, sometimes terminating fatally, developed after the patient had convalesced from the initial fever and had returned to work. It is a point of additional interest to note that animals may be affected as well as man. Dana, in the *Boston Medical and Surgical Journal* for 1905, has recorded an epidemic of the disease in which paralysis attacked animals in the same district as that in which the human cases occurred.

All these facts point to a bacterial cause of the disease. The evidence upon this point has been ably sifted at the Middlesex Hospital, and it is concluded that of all the researches upon the subject that have been published, those of Geirsvold are the most thorough and convincing. Having found sources of fallacy in the results published by some other observers, they say*: "The results obtained by Geirsvold, on the other hand, were consistent, and afford the strongest presumptive evidence that he was successful in isolating a diplococcus which stood in causative relation to the disease under investigation. Geirsvold examined the cerebro-spinal fluid obtained either by lumbar puncture during life or after death.

* Geirsvold: "Tidsskrift for den Norske Lægeforening."
† Pasteur: "Transactions of the Clinical Society of London," Vol. XXX., No. 20, 190.
‡ Pasteur, Foulerton and MacCormac: "Archives of the Middlesex Hospital," Vol. XII., p. 13.

* *Loc. cit.*, p. 24.

. . . It does not appear how many cases were examined, but in twelve cases Geirsvold obtained in pure culture a diplo- or tetro-coccus, which readily produced chains of four or six elements when growing in nutrient broth." The organism retained the stain by Gram's method. The organism proved to be pathogenic both for mice and for rabbits, and paralysis was a common but not a constant result of inoculating these animals with it. It seems clear that we must no longer regard acute anterior poliomyelitis as a disease. Like jaundice, it is only a symptom. One cause of it—and perhaps its chief cause—is a specific infective disease which has at present no name.

